# Resistance of *Anopheles gambiae* sensu lato to Pirimiphos-methyl Insecticide in Kakamega County, Highlands of Western Kenya

**DOI:** 10.4314/ahs.v22i1.68

**Published:** 2022-03

**Authors:** Nicholas Kitungulu, Bernard Guyah, Mark Webale, Nathan Shaviya, Maxwell Machani, David Mulama, Bryson Ndenga

**Affiliations:** 1 Maseno University, Department of Biomedical Sciences & Technology; Masinde Muliro University of Science and Technology, Department of Biological sciences; 2 Masinde Muliro University ofScience and Technology, Department of Biological sciences; 3 Kirinyaga University, Department of Health Sciences; 4 Masinde Muliro University of Science and Technology, Department of Medical Laboratory Sciences; 5 Kenya Medical Research Institute, Centre for Global Health Research

**Keywords:** *Anopheles gambiae* s.l, G119S mutation, Pirimiphos-methyl, Resistance

## Abstract

**Background:**

Insecticide treated bed nets and Indoor residual spraying remains the principal interventional malaria control strategies. To achieve malaria disease eradication, vector control programmes that monitor insecticide resistance profiles are necessary.

**Objective:**

The study evaluated pirimiphos-methyl susceptibility of *Anopheles gambiae* sensu lato in Kakamega County, western Kenya.

**Methods:**

Adult *Anopheles gambiae* sensu lato mosquitoes were assayed using World Health Organization tube bioassay against 0.25% pirimiphos-methyl. Susceptible and non-susceptible populations were characterized to species-level using Polymerase Chain Reaction. Susceptible and resistant mosquitoes were further subjected to G119S Acetylcholisterase (ace 1R) mutation detection.

**Results:**

*Anopheles arabiensis* was the predominant species in all study population Mumias east (62%), Malava (68%), Ikolomani (77%) and Lurambi (82%). Results showed phenotypic susceptibility to pirimiphos-methyl. Mortality was low in Mumias east (80.6%) and high in Lurambi (89.0%). G119S mutations ranged from 3.0% to 8.9% in *Anopheles arabiensis* whereas G119S mutations were relatively low ranging from 0.0% to 3.1% in *Anopheles gambiae* s.s populations. Study populations tested were consistent with Hardy-Weinberg equilibrium (P>0.05).

**Conclusion:**

We observed pirimiphos-methyl resistance in Anopheles arabiensis and *Anopheles gambiae* s.s. study populations. Results showed G119S mutation in resistance population. Resistance monitoring and management are urgently required.

## Background

Malaria disease transmission is a great public health concern globally 1. The disease remains a major cause of morbidity and mortality in sub-Saharan Africa 2. Kakamega County is among counties with high stable malaria infections 3,4. *Anopheles gambiae* sensu stricto (s.s) and *An. arabiensis* are the major malaria vectors in the highlands of western Kenya 5. Indoor residual spraying (IRS) and insecticidal-treated nets (ITNs) are the major important tools in malaria disease elimination in sub-Saharan Africa 6. However, Anopheles mosquitoes have demonstrated reduced susceptibility to at least one public health insecticide recommended by World health Organization (WHO) 7,8 jeopardizing malaria control efforts.

The effectiveness of vector control strategy relies majorly on knowledge of vector species and their resistance to insecticides. Pyrethroids are the only recommended class of insecticide for use in both IRS and ITNs possibly because of limited effects to users 9 while carbamates, organochlorides, anorganophosphates are only applied in IRS 10. Pyrethroids and organochlorines are neurotoxins that act by prolonging the voltage-gated sodium channel activation whereas the organophosphates act on the central nervous system by inhibiting the expression of acetylcholinesterase enzyme 11,12. Pyrethroids resistance in malaria vectors has mostly been studied in the major malaria vector *An. gambiae* s.l of Africa 12–17. The two major mechanisms associated with insecticide resistance are; a) Target site insensitivity in the sodium channel, causing a change in affinity between the insecticide and its binding site; b) metabolic detoxification of insecticides before reaching the target site 18. Several studies have confirmed the existence of pyrethroids and organochlorines insecticides resistance in some parts of western Kenya, with knockdown resistance (kdr) mutations at position 1014 in the sodium channel gene being commonly reported 8,19,20. However, there is limited information on the status of pirimiphos-methyl insecticide resistance amongst the *An. gambiae* s.l complex in Kenya.

Owing to the increased resistance of malaria vectors to available insecticides, several alternative insecticides have been developed to control and eliminate mosquitoes. Pirimiphos-methyl (p-methyl) an organophosphate insecticide, has a fast action with minimal toxicity in both humans and environment 21. The application of pirimiphos-methyl has been effective in Killing malaria vectors 22,23. This however, could be because of its low eminence and high vapor pressure 24,25. Indoor residual spraying controlled trial studies indicate the presence of high levels of toxicity of p-methyl to mosquitoes 26,27. This led World Health Organization Pesticides Evaluation Scheme (WHOPES) to recommend use of p-methyl application as IRS for malaria vector control 28. In Africa, resistance to insecticides such as pyrethroids and Dichloro-diphenyltrichloroethane (DDT) has frequently been associated with pesticide usage in agricultural farms 29,30. For instance An. gambiae females lay their eggs in breeding sites around agricultural settings 29, this consequently suggests that mosquito larvae may undergo selection pressure from agricultural pesticides favouring the occurrence of resistance to insecticides by malaria vectors 31–34. This study evaluated pirimiphos-methyl insecticides susceptibility status among Anopheles gambiae sensu lato in four randomly selected sub-counties in Kakamega County, highlands of western Kenya.

## Methods

### Study area

**Figure 1:**
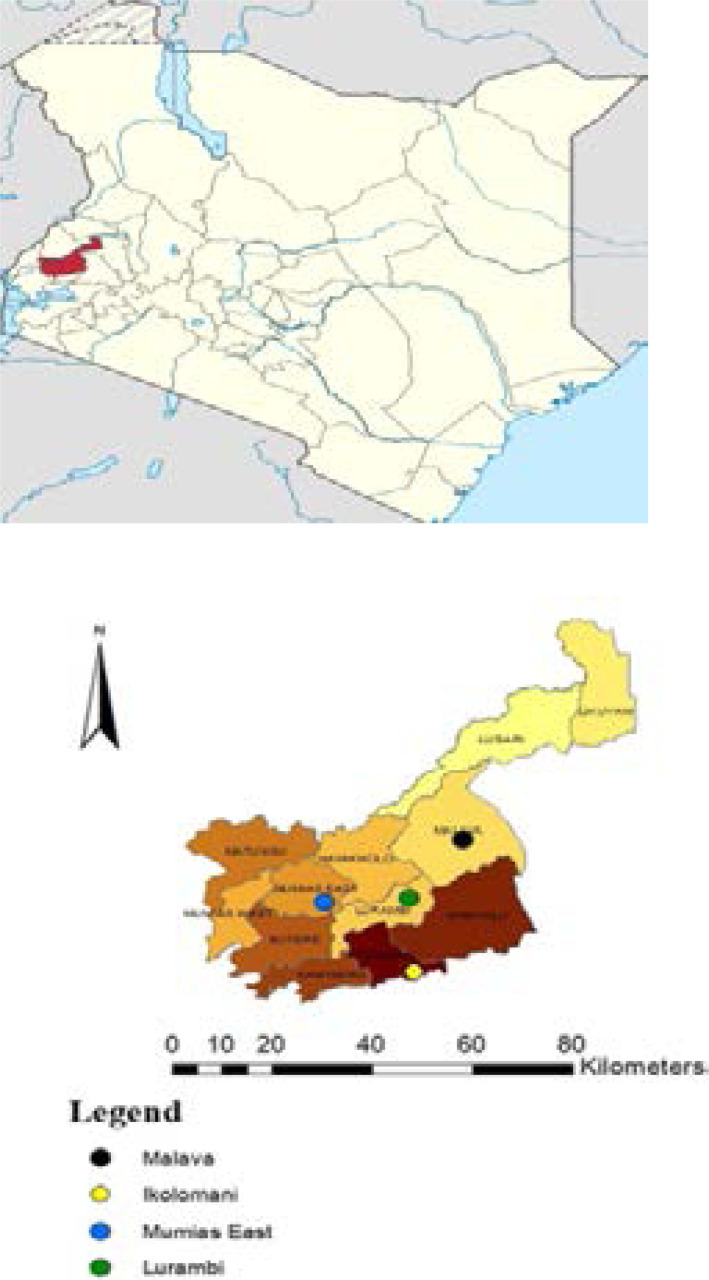
Map showing the study area (Kakamega County), western Kenya

A cross-sectional survey was conducted in four highlands located in Kakamega County of western Kenya namely; Ikolomani; (E:00.16556, N:034.73194), Mumias East; (E:00.34120, N:034.54727), Lurambi; (E:00.31806, N:034.75222) and Malava; (E:00.32957, N:034.74701) (Figure 1). The study sites experienceseasonal malaria transmission, with high peaks occurring at the end of long (early April to late July) and short (October to November) rainy seasons. The main economic activity in the study areas is agriculture. The main food crops are sugarcane, maize farming and short-season farming such as vegetable farming. Multiple classes of pesticides such as organophosphate, pyrethroids and carbamates are used in controlling crop pests. The used pesticides end up seeping into the breeding habitats exposing the mosquito population to chemical residues.

### Mosquito sampling and rearing

*Anopheles gambiae* s.l larvae were sampled in their natural breeding habitats using a standard 350-milliliter dipper from October 2018 to March 2019. Mosquito larvae were randomly collected per habitat and identified morphologically to Anopheline species using a key developed by Gillies 35. Collected larvae were pooled together in each study site then transferred to an insectary at Masinde Muliro University of Science and Technology for rearing at 80 ± 10% RH, 25 ± 2 °C, 12h:12h light/dark cycle. Larvae were cultured in spring water in small trays and fed on Tetramin™ baby fish food (TetraMin tropical flakes, Blacksburg, VA, USA) every day. Individual pupated larvae were collected and transferred into a 1ft square cage containing a net. Emerged adults were prvided with 10% sugar solution until they were 3 to 5 days old. The Kisumu strain of *An. gambiae* sensu stricto, a reference strain susceptible to all insecticides, was reared simultaneously under the same conditions and used as a control for insecticide bioassays..

### WHO susceptibility bioassay

Emerged Adult females of *An. gambiae* s.l aged 3–5 days were tested for susceptibility to pirimiphos-methyl using the standard WHO tube bioassay (36) with 0.25% diagnostic dose as recommemded by WHO. For each population, 100 active female mosquitoes were tested. Paraffin oil-treated papers without insecticide were used as control. The knockdown time was recorded every 10 minutes within one hour exposure period. After 1 hour of exposure to the diagnostic concentration of pirimiphos-methyl (0.25%), experimental mosquitoes were transferred to recovery cups and maintained on 10% sucrose solution for 24 hours. Mortality was recorded at 24 hours post-exposure time. Knocked down mosquitoes after 1 hour and those that were alive after the 1 hour exposure period and still surviving 24 hours later were collected and stored individually in 90% alcohol for further molecular analysis. A susceptible mosquito was defined as a mosquito that died within 24 hours of recovery period or knocked down during the 1 hour exposure time while a resistant mosquito was referred as a mosquito that survived after the end of the 24 hours recovery period.

### Molecular characterization of Anopheles gambiae s.l

Dead and Survived mosquitoes preserved after the bioassays were identified to species level using species-specific polymerase chain reaction (PCR) (PCR Model FTC 3105D, TECHNE, DUXFORD CAMBRIDGE, UK) assay previously described by Scott 37. Deoxyribonucleic acid (DNA) was extracted from legs and wings of each mosquito using the ethanol precipitation method (38). The PCR technique was used to distinguish between the two sibling species of Anopheles gambiae s.l species complex native to western Kenya namely; *An. gambiae* s.s and An. arabiensis 38.

### Molecular detection of G119S Ace-1R mutation

Dead and survived samples of *An. anopheles* s.l post exposure were subjected to G119S Ace-1R analysis. The polymerase chain reaction was done using a protocol described by Essandoh and collaborators39. In summary, each reaction constituted a volume of 50ul containing 10 picomoles of each primers Ex2Agdir1 (5′ AGG TCA CGG TGA GTC CGT ACG A 3′) and Ex4Agrev2 (5′ AGG GCG GAC AGC AGA TGA AGC GA 3′), 10mM dNTPs, ddH2O, 5X HF Phusion buffer and 1ul of Phusion Taq polymerase (Fermentas). The cycle parameters were: 1 cycle at 98°C for 4 mins, followed by 35 cycles of 98°C for 30 sec, 64°C for 15 sec and 72°C for 30 secs with final extension at 72°C for 5 mins

### Data management and analysis

Collected data were entered in MS-Excel spreadsheet, checked, cleaned after which it was coded and imported into SPSS version 19.0 (IBM SPSS Statistics for Windows, Version 19.0. Armonk, NY: IBM Corp) for analysis. The mortality rates of mosquitoes in the standard WHO tube resistance bioassay were calculated and adjusted using Abbott's formula 40. Susceptibility status of mosquito population was classified according to the WHO criteria (98–100 % mortality indicates susceptibility, 90–97 % mortality suggests the possibility of resistance that needs to be confirmed, and <90 % mortality suggests resistance) 36. Heterozygous and homozygous mutation rates of Ace-1R gene loci (G119S) were calculated. To determine if these genotypes were under selection, the Hardy-Weinberg equilibrium test for Ace-1R genotypes was performed, and the Chisquare test used to determine the significance of the departure from Hardy-Weinberg equilibrium.

### Ethics Statement

This study was approved by the Maseno University Ethical Review Committee (MUERC) under the scientific steering committee (MUERC/0061/18). The current study did not involve endangered or protected species.

## Results

### Susceptibility status of the study populations

**Figure 2:**
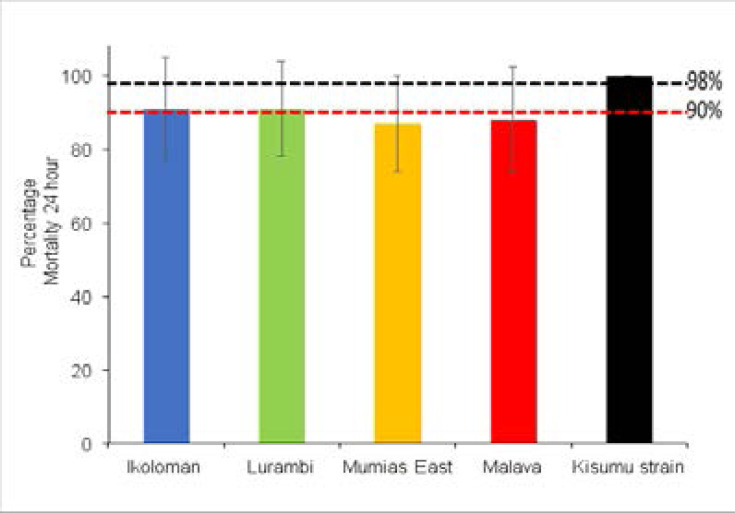
Susceptibility status of *Anopheles gambiae* s.l to pirimiphos-methyl insecticide Mortality rates of *Anopheles gambiae* s.l in the standard WHO tube insecticide susceptibility test. Standard diagnostic dosage of 0.25% pirimiphos-methyl.

Mosquito populations collected from Mumias east and Malava showed phenotypic resistance to 0.25% p-methyl with mortality rates of 87% and 88% respectively (Figure 2). There was suspected insecticide resistance in Ikolomani and Lurambi mosquito populations with both populations recording 91% mortality rate. At the species level (Table 1), the study observed phenotypic resistance in Anopheles arabiensis with mortality ranging from 80.6% to 89.0% in all the study populations. On the other hand, *Anopheles gambiae* s.s population from Ikolomani showed resistance to p-methyl (86.9%). There was suspected p-methyl resistance (90.6%) in Malava mosquito population while Mumias east showed mortality of 97.4% whereas the Lurambi population was 100% susceptible. WHO tube bioassay showed tat Kisumu strain was 100% susceptible, this confirms the quality of the insecticide-impregnated papers used.

**Table 1:** Susceptibility 1 status of *Anopheles gambiae* s.l to pirimiphos-methyl insecticide

*Anopheles gambiae* s.l								
Study site	*Anopheles arabiensis*				*Anopheles gambiae s.s*			
	N	Mortality (%)	95% Cl	Status	N	Mortality %	95% Cl	Status
Ikolomani	77	81.8	73.2–90.4	R	23	86.9	73.2100	R
Lurambi	82	89.0	82.3–958	R	18	100.0	N/A	S
Mumias East	62	80.6	70.8–90.5	R	38	97.4	92.3100	S
Malava	68	86.7	78.7–94.8	R	32	90.6	80.5100	SR

Key: N, Sample size; %, proportion in percentage; s.s, sensu stricto; CL, confidence interval; R, resistance; S, susceptible; SR, suspected resistance; N/A, not applicable.

### Anopheles gambiae s.l sibling species characterization

All the Anopheles gambiae s.l exposed to p-methyl were subjected for sibling species identification using PCR. The two species of the An. gambiae complex in Kakamega County of western highlands, that is; An. arabiensis and An. gambiae s.s were found to be sympatric in the four surveyed sites with different frequencies. Anopheles arabiensis was the predominant species in all study population Mumias east (62%), Malava (68%), Ikolomani (77%) and Lurambi (82%) (Table 1).

### Ace-1R G119S genotyping

G119S mutations ranged from 3.0% to 8.9% in Anopheles arabiensis dominant populations, whereas G119S mutations were relatively low ranging from 0.0% to 3.1% in Anopheles gambiae s.s populations. Additionally, most of the mutations were heterozygous in Anopheles arabiensis (Table 2). No mutations were observed in Lurambi population. Hardy-Weinberg analysis showed that for the four An. gambiae s.s populations tested, only one population (Lurambi) had zero percent mutation whereas only Mumias East had only one rear homozygous G119S in An. arabiensis species (Table 2). All the populations tested were consistent with Hardy-Weinberg equilibrium as the populations had P>0.05. After exposure to pirimiphos-methyl, the G119S mutation was also found to be present in survivors (Table 3). The survival of Anopheles gambiae (s.l) populations from the three surveyed areas was associated with the presence of the mutation.

**Table 2:** Genotype and alleles frequencies of *Ace-1^R^* (locus 119) at the four study sites

Study site	***Anopheles arabiensis***						
	Sample size (N)	GG	GS	SS	Frequency %	χ^2^	*P*-value
Ikolomani	77	69	8	0	5.2	0.231	0.631
Lurambi	82	77	5	0	3.0	0.081	0.773
Mumias East	62	52	9	1	8.9	0.647	0.421
Malava	68	63	5	0	3.7	0.099	0.753
***Anopheles gambiae* s.s**							
	Sample size (N)	GG	GS	SS	Frequency %	χ^2^	*P*-value
Ikolomani	23	22	1	0	2.2	0.011	0.915
Lurambi	18	18	0	0	0.0	N/A	N/A
Mumias East	38	37	1	0	1.3	0.007	0.934
Malava	32	30	2	0	3.1	0.033	0.855

Key: N is sample size; GG represent wild type genotype; GS represent heterozygous mutant; SS represent rear homozygous mutant; χ^2^Chi-squared test; P is significant level and Frequency is the mutation allele frequencies in percentage. Note, N/A means not applicable while χ^2^ and *P*-value are the results of Hardy Weinberg equilibrium test.

**Table 3:** Allelic frequencies distribution of G119S genotype of *Anopheles gambiae* s.l populations in resistance and susceptible mosquitoes post p-methyl insecticide exposure

Study site	N	Resistance (%)		Susceptibility (%)	
Ikolomani	100	Wild type	Mutant	Wild type	Mutant
		8.0	9.0	83.0	NM

Lurambi	100	4.0	5.0	91.0	NM

Mumias East	100	3.0	10.0	87.0	NM

Malava	100	5	7	88	NM

Key: N is sample size; % is proportion in percentage; NM, no mutant

## Discussion

The present study evaluated the resistance status of *Anopheles gambiae* sensu lato to pirimiphos-methyl insecticide. We observed that the predominant species in our study population was *Anopheles arabiensis*. This corroborates with similar studies in some parts of western Kenya and East African^13^,^41^–^44^. Phenotypic susceptibility tests revealed that *An. arabiensis* populations showed reduced mortality to p-methyl insecticide, according to the susceptibility threshold level 10. This agrees with a previous study that reported the emergence of resistance among *An. arabiensis* to p-methyl in some regions of Ethiopia^45^. Similarly, the findings mirror previous studies in Tanzania that reported low frequency of p-methyl resistance^23^. Additionally, resistance was observed in An. gambiae s.s from Ikolomani sub-county study population and suspected resistance in Malava sub-county. The observed scenario in this particular species may have been as a result of fewer numbers of *An. gambiae* s.s mosquitos in study populations. The observed resistance could be due to the application of agrochemicals pesticides and herbicides in agricultural farms that belongs to organophosphates class of insecticides/pesticides^33^,^39^,^46^–^48^. It is approximated that 60% of farmers in western Kenya use pesticides for pest control^49^. Consequently, during the application of pesticides, residues seep into mosquito breeding habitats. This might have driven the observed emergence of p-methyl insecticide resistance in *An. gambiae* s.l^54,55^. However, the findings of this study are inconsistent with previous studies in Migori County that reported 100% susceptibility of An. gambiae s.l to p-methyl^27^,^56,57^. Migori County is one of the counties where IRS is in use. This implies that continuous application of insecticide is likely to be associated with resistance in mosquitoes^58^.

The current study showed the presence of the G119S mutation in An. gambiae s.l across the study populations. This may be the first study to report such findings in western Kenya. Allelic frequencies of the G119S mutation were higher in An. arabiensis than in An. gambiae s.s. Related observations were reported in Ghana, Burkina Faso and Ethiopia^59,60,^^49^. Association between ace 1 mutation frequencies and observed phenotypic p-methyl resistance profiles in the surveyed sites; the study found that all resistant mosquitoes had the resistant allele (S) in its heterozygous form with only one mosquito (An. arabiensis) from Mumias east having a rear homozygous (SS). Some studies have reported that homozygous resistant individuals are most likely to die during pupation than susceptible individuals61, 62, 63. The study observed mutations in phenotypically resistance mosquitoes whereas some of the resistant mosquitoes did not exhibit the mutations. Possibly suggestion of other existence resistance mechanisms that may be driving resistance of Anopheles gambiae s.l. Therefore, the low frequency of G119S mutations is an alert for an exigent need to create measures to avoid allele fixation in the mosquito population

## Conclusion

The study showed pirimiphos-methyl resistance in *Anopheles arabiensis* and *Anopheles gambiae* s.s. study populations. Results indicate G119S mutation in resistance population. Furthermore, there were wildtype phenotypically resistant populations that did not harbor G119S mutations suggesting other resistance mechanisms. Resistance monitoring and management of insecticide resistance are urgently required.

## Declarations

### Availability of data and materials

The data used to support the findings of this study are included within the article.

## References

[1] WHO (2019). Global malaria report 2019.

[2] Wolrd Health Organization (2020). World malaria report 2020: 20 years of global progress and challenges.

[3] Kenya Malaria Indicator Survey 2015.

[4] Bashir IM, Nyakoe N, van der Sande M (2019). Targeting remaining pockets of malaria transmission in Kenya to hasten progress towards national elimination goals: an assessment of prevalence and risk factors in children from the Lake endemic region. Malar J.

[5] Okara RM, Sinka ME, Minakawa N, Mbogo CM, Hay SI, Snow RW (2010). Distribution of the main malaria vectors in Kenya. Malar J.

[6] Mathias DK, Ochomo E, Atieli F, Ombok M, Bayoh MN, Olang G (2011). Spatial and temporal variation in the kdr allele L1014S in Anopheles gambiae s.? and phenotypic variability in susceptibility to insecticides in Western Kenya. Malar J.

[7] Ochomo E, Bayoh NM, Kamau L, Atieli F, Vulule J, Ouma C (2014). Pyrethroid susceptibility of malaria vectors in four Districts of western Kenya. Parasit Vectors.

[8] Ochomo E, Subramaniam K, Kemei B, Rippon E, Bayoh NM, Kamau L (2015). Presence of the knockdown resistance mutation, Vgsc-1014F in Anopheles gambiae s.l and An. Arabiensis in western Kenya. Parasit Vectors.

[9] Zaim M, Aitio A, Nakashima N (2000). Safety of pyrethroid-treated mosquito nets. Med Vet Entomol.

[10] World Health Organization (2013). WHO recommended insecticides for indoor residual spraying against malaria vectors: WHO Pesticides Evaluation Scheme (WHOPES).

[11] Davies TGE, Field LM, Usherwood PNR, Williamson MS (2007). DDT, pyrethrins, pyrethroids and insect sodium channels. IUBMB Life.

[12] Soderlund DM, Bloomquist JR (1989). Neurotoxic actions of pyrethroid insecticides. Annu Rev Entomol.

[13] Mathias DK, Ochomo E, Atieli F, Ombok M, Bayoh MN, Olang G (2011). Spatial and temporal variation in the kdr allele L1014S in Anopheles gambiae ss and phenotypic variability in susceptibility to insecticides in Western Kenya. Malar J.

[14] Edi CVA, Koudou BG, Jones CM, Weetman D, Ranson H (2012). Multiple-Insecticide Resistance in Anopheles gambiae Mosquitoes, Southern Côte d'Ivoire. Emerg Infect Dis.

[15] Kawada H, Dida GO, Ohashi K, Komagata O, Kasai S, Tomita T (2011). Multimodal pyrethroid resistance in malaria vectors, Anopheles gambiae s.s, Anopheles arabiensis, and Anopheles funestus s.s in western Kenya. Plos One.

[16] Kawada H, Futami K, Komagata O, Kasai S, Tomita T, Sonye G (2011). Distribution of a knockdown resistance mutation (L1014S) in Anopheles gambiae s.s and Anopheles arabiensis in Western and Southern Kenya. Plos One.

[17] Ranson H, Abdallah H, Badolo A, Guelbeogo WM, Kerah-Hinzoumbé C, Yangalbé-Kalnoné E (2009). Insecticide resistance in Anopheles gambiae: data from the first year of a multi-country study highlight the extent of the problem. Malar J.

[18] Hemingway J, Hawkes NJ, mccarroll L, Ranson H (2004). The molecular basis of insecticide resistance in mosquitoes. Insect Biochem Mol Biol.

[19] Hemming-Schroeder E, Strahl S, Yang E, Nguyen A, Lo E, Zhong D (2018). Emerging Pyrethroid Resistance among Anopheles arabiensis in Kenya.

[20] Machani MG, Ochomo E, Amimo F, Kosgei J, Munga S, Zhou G (2020). Resting behaviour of malaria vectors in highland and lowland sites of western Kenya: Implication on malaria vector control measures. PLos One.

[21] Fukuto TR (1990). Mechanism of action of organophosphorus and carbamate insecticides. Environ Health Perspect.

[22] Haji KA, Thawer NG, Khatib BO, Mcha JH, Rashid A, Ali AS (2015). Efficacy, persistence and vector susceptibility to pirimiphos-methyl (Actellic® 300CS) insecticide for indoor residual spraying in Zanzibar. Parasit Vectors.

[23] Oxborough RM, Kitau J, Jones R, Feston E, Matowo J, Mosha FW (2014). Long-lasting control of Anopheles arabiensis by a single spray application of micro-encapsulated pirimiphos-methyl (Actellic® 300 CS). Malar J.

[24] Patsoula E, Samanidou-Voyadjoglou A, Spanakos G, Kremastinou J, Nasioulas G, Vakalis NC (2006). Molecular and morphological characterization of Aedes albopictus in northwestern Greece and differentiation from Aedes cretinus and Aedes aegypti. J Med Entomol.

[25] Tabbabi A, Daaboub J, Laamari A, Cheikh RB, Ben CH (2017). Pirimiphos-Methyl Resistance Status of Field Populations of Culex pipiens (Diptera: Culicidae) From Grand Tunis Area, Northeast Tunisia. Hered Genet.

[26] Nasir SM, Ahmad N, Shah MA, Azam CM (1982). A large-scale evaluation of pirimiphos-methyl 25% WP during 1980-1981 for malaria control in Pakistan. J Trop Med Hyg.

[27] Abong'o B, Gimnig JE, Torr SJ, Longman B, Omoke D, Muchoki M (2020). Impact of indoor residual spraying with pirimiphos-methyl (Actellic 300CS) on entomological indicators of transmission and malaria case burden in Migori County, western Kenya. Sci Rep.

[28] World Health Organization (2013). Report of the sixteenth WHOPES working group meeting: WHO/HQ, Geneva, 22-30 July 2013: review of Pirimiphos-methyl 300 CS, Chlorfenapyr 240 SC, Deltamethrin 62.5 SCPE, Duranet LN, Netprotect LN, Yahe LN, Spinosad 83.3 Monolayer DT, Spinosad 25 Extended release GR.

[29] Diabate A, Baldet T, Chandre F, Akoobeto M, Guiguemde TR, Darriet F (2002). The role of agricultural use of insecticides in resistance to pyrethroids in Anopheles gambiae sl in Burkina Faso. Am J Trop Med Hyg.

[30] Yadouleton AWM, Asidi A, Djouaka RF, Braïma J, Agossou CD, Akogbeto MC (2009). Development of vegetable farming: a cause of the emergence of insecticide resistance in populations of Anopheles gambiae in urban areas of Benin. Malar J.

[31] Akogbéto MC, Djouaka RF, Kindé-Gazard DA (2006). Screening of pesticide residues in soil and water samples from agricultural settings. Malar J.

[32] Matowo J, Kulkarni MA, Mosha FW, Oxborough RM, Kitau JA, Tenu F (2010). Biochemical basis of permethrin resistance in Anopheles arabiensis from Lower Moshi, north-eastern Tanzania. Malar J.

[33] Nkya TE, Akhouayri I, Poupardin R, Batengana B, Mosha F, Magesa S (2014). Insecticide resistance mechanisms associated with different environments in the malaria vector Anopheles gambiae: a case study in Tanzania. Malar J.

[34] Nkya TE, Akhouayri I, Kisinza W, David J-P (2013). Impact of environment on mosquito response to pyrethroid insecticides: facts, evidences and prospects. Insect Biochem Mol Biol.

[35] Gillies MT, De Meillon B (1968). The anophelinae of Africa south of the Sahara (Ethiopian zoogeographical region). Anophelinae Afr South Sahara Ethiop Zoogeographical Reg.

[36] World Health Organization (2016). Test procedures for insecticide resistance monitoring in malaria vector mosquitoes.

[37] Scott JA, Brogdon WG, Collins FH (1993). Identification of single specimens of the Anopheles gambiae complex by the polymerase chain reaction. Am J Trop Med Hyg.

[38] Collins FH, Mendez MA, Rasmussen MO, Mehaffey PC, Besansky NJ, Finnerty V (1987). A ribosomal RNA gene probe differentiates member species of the Anopheles gambiae complex. Am J Trop Med Hyg.

[39] Essandoh J, Yawson AE, Weetman D (2013). Acetylcholinesterase (Ace-1) target site mutation 119S is strongly diagnostic of carbamate and organophosphate resistance in Anopheles gambiae s.s and Anopheles coluzzii across southern Ghana. Malar J.

[40] Abbott WS (1925). A method of computing the effectiveness of an insecticide. J Econ Entomol.

[41] Tonnang HE, Kangalawe RY, Yanda PZ (2010). Predicting and mapping malaria under climate change scenarios: the potential redistribution of malaria vectors in Africa. Malar J.

[42] Ototo EN, Mbugi JP, Wanjala CL, Zhou G, Githeko AK, Yan G (2015). Surveillance of malaria vector population density and biting behaviour in western Kenya. Malar J.

[43] Bayoh MN, Mathias DK, Odiere MR, Mutuku FM, Kamau L, Gimnig JE (2010). Anopheles gambiae: historical population decline associated with regional distribution of insecticide-treated bed nets in western Nyanza Province, Kenya. Malar J.

[44] Kitau J, Oxborough RM, Tungu PK, Matowo J, Malima RC, Magesa SM (2012). Species shifts in the Anopheles gambiae complex: do llins successfully control Anopheles arabiensis. Plos One.

[45] Messenger LA, Shililu J, Irish SR, Anshebo GY, Tesfaye AG, Ye-Ebiyo Y (2017). Insecticide resistance in Anopheles arabiensis from Ethiopia (2012–2016): a nationwide study for insecticide resistance monitoring. Malar J.

[46] Djogbénou L, Dabiré R, Diabaté A, Kengne P, Akogbéto M, Hougard JM (2008). Identification and geographic distribution of the ACE-1R mutation in the malaria vector Anopheles gambiae in south-western Burkina Faso, West Africa. Am J Trop Med Hyg.

[47] Nkya TE, Poupardin R, Laporte F, Akhouayri I, Mosha F, Magesa S (2014). Impact of agriculture on the selection of insecticide resistance in the malaria vector Anopheles gambiae: a multigenerational study in controlled conditions. Parasit Vectors.

[48] Philbert A, Lyantagaye SL, Nkwengulila G (2014). A review of agricultural pesticides use and the selection for resistance to insecticides in malaria vectors. Adv Entomol.

[49] Osano O, Nzyuko D, Tole M, Admiraal W (2003). The fate of chloroacetanilide herbicides and their degradation products in the Nzoia Basin, Kenya. AMBIO J Hum Environ.

[50] Riveron JM, Watsenga F, Irving H, Irish SR, Wondji CS (2018). High Plasmodium infection rate and reduced bed net efficacy in multiple insecticide-resistant malaria vectors in Kinshasa, Democratic Republic of Congo. J Infect Dis.

[51] Philbert A, Lyantagaye SL, Nkwengulila G (2014). A review of agricultural pesticides use and the selection for resistance to insecticides in malaria vectors. Adv Entomol.

[52] Mbepera S, Nkwengulila G, Peter R, Mausa EA, Mahande AM, Coetzee M (2017). The influence of age on insecticide susceptibility of Anopheles arabiensis during dry and rainy seasons in rice irrigation schemes of Northern Tanzania. Malar J.

[53] Echodu R, Anena J, Iwiru T, Mireji P, Malinga GM, Opiyo EA (2020). High level of resistance in the mosquito Anopheles arabiensis to pyrethroid insecticides from low malaria transmission zone of Moroto district, Karamoja region, Uganda: Implication for malaria vector control.

